# A Facile Green Fabrication and Characterization of Cellulose-Silver Nanoparticle Composite Sheets for an Antimicrobial Food Packaging

**DOI:** 10.3389/fnut.2021.778310

**Published:** 2021-12-03

**Authors:** Seongyoung Kwon, Wooseok Lee, Jung Wook Choi, Nattinee Bumbudsanpharoke, Seonghyuk Ko

**Affiliations:** ^1^Laboratory of Nano-Enabled Packaging and Safety, Department of Packaging, Yonsei University, Wonju, South Korea; ^2^Agency for Korea National Food Cluster, Ministry of Agriculture, Food and Rural Affairs, Iksan, South Korea; ^3^Department of Packaging and Materials Technology, Faculty of Agro-Industry, Kasetsart University, Bangkok, Thailand

**Keywords:** green synthesis, cellulose fiber, silver nanoparticles, antimicrobial activity, food packaging

## Abstract

The present study focused on a facile and green approach for the one-step synthesis of silver nanoparticles (AgNPs) embedded in hard wood bleached kraft fiber. The hydroxyl groups on the cellulose chain induced ionic silver reduction with additional hydrothermal energy, allowing for the *in situ* formation and deposition of AgNPs on the cellulose fiber. The white color of the bleached fiber transformed to yellow due to the formation of AgNPs. UV-Vis spectroscopy, scanning electron microscopy, and X-ray photoelectron spectroscopy revealed that the AgNPs were uniformly distributed across the surface of the obtained cellulose fibers. The results indicated that the formation and distribution of AgNPs on surface of cellulose fibers was significantly influenced by the amount and concentration of silver nitrate (AgNO_3_). The antimicrobial activity of the cellulose-AgNP composite sheet against *Escherichia coli* was found to be inhibiting. These findings imply that cellulose-AgNP composite sheets can be feasibly used as antimicrobial paper for food packaging.

## Introduction

Nanobiotechnology is one of the most active research areas in modern material science. A variety of bioresources have been used as green chemicals in the biosynthesis of noble metal nanoparticles such as silver (1–3), gold (4–6), and platinum (7–9). These efforts directed toward achieving environmentally friendly technology are gaining tremendous traction. The reactive hydroxyl and carboxyl groups from natural bio-derived materials have been used as environmentally benign reducing agents in this approach, replacing harmful reductive organic species such as sodium borohydride (NaBH4), hydrazine, and dimethylformamide (DMF), which pose potential environmental and biological risks (10, 11). Cellulose, an abundant natural polymer, is employed as an excellent green reducing agent candidate owing to its mild reductive ability, which is due to a large number of hydroxyl and ether groups on its polysaccharide chains (12). In general, the green synthesis of metal nanoparticles necessitates the addition of additional energy to activate the system; thus, several sources of external energy have been incorporated into the process. Sedighi et al. (13) used thermal energy to improve the reduction power of cotton fabric for the synthesis of copper and copper oxide nanoparticles. Fernandez et al. (14) prepared a silver nanocomposite with cellulose nanofibers *via* the thermal and UV radiation treatment. Nadagouda and Varma (15) and Chen et al. (16) demonstrated the use of microwave-assisted green synthesis for transition metal nanoparticles (i.e., copper, silver, indium, and iron) by incorporating carboxymethyl cellulose sodium as a reducing and stabilizing reagent in the reaction. The direct synthesis or growth of metal nanoparticles on cellulosic materials is of interest for many applications, including fabrics and textiles (17–19), catalysis (20–22), biomedical device (23–25), and active packaging (26–28).

The packaging industry has evolved significantly in the last two decades. The changing dynamics of consumer demand and market trends have compelled the food industry to seek superior product quality and safety. As a result, active packaging is becoming increasingly important. Several types of active packaging technology are currently being used in the food industry, including control and release systems for active ingredients, antioxidant and antimicrobial materials, and desired gas scavenging and generation systems (29, 30). These technologies involve chemical, biological, and physical actions that alter the interactions between the products, packaging, and environment, thereby extending the shelf life (31). In order to modify the gas composition within the packaging headspace, antioxidant and antibacterial substrates are usually absorbent pads, sachets, or coating materials comprised of volatile active chemicals (i.e., ethanol, chlorine dioxide, sulfur dioxide, and essential oils). Non-volatile active substances (such as organic acid and nanoparticles) are typically placed under the food and function *via* direct contact to the target chemicals or microorganism (32). Among all types of active packaging, antibacterial packaging is expected to grow over the next decade, aided by the introduction of new materials and the integration of other emerging technologies such as nanotechnology or biotechnology (33–35).

Innovative research in the area of active packaging and containers, focusing on antimicrobial properties of silver nanoparticles (AgNPs), is crucial to the growth of food and beverage industries (36). It has been proposed that AgNP is less toxic to human cells than to bacterial cells, owing to the relatively high acute toxicity to microorganisms. Furthermore, AgNPs have broad antimicrobial activity against Gram-positive and Gram-negative bacteria, fungi, and viruses (37). Although the antimicrobial mechanism is unclear, it is believed that microbial inhibition is caused by both the AgNPs and the discharged Ag ions (38). AgNPs can be a coating material on a fruit peel (39) or packaging surface for food wrapper (40, 41), as well as an antibacterial additive substance directly integrated in a polymer matrix (42, 43). They have the potential to increase the shelf life of food products by preventing the growth of food spoilage microorganisms during transportation and storage (44, 45). Furthermore, the *in vitro* research for AgNPs migration from commercial plastic food packaging and containers revealed that AgNPs can be released from plastics and the amount released was sufficient to kill microorganism (46–48). Several formats of AgNP fabrication for active packaging have been developed, including absorbent pads (14, 49), wrapping film (50, 51), and paper sheet (52–54) with simple techniques by directly blending or coating the AgNP colloids or powders to a packaging material (55, 56). However, for food preservation with active packaging, it is difficult to use colloidal AgNPs as antimicrobial agents because of the challenges in coagulation, aggregation, and fabrication (42, 57). To address these issues, classic methods of direct immobilization or encapsulation of AgNPs on supporting matrices are used.

In this study, the synthesis method was adapted from our previous research (4, 26), which was for the green synthesis of gold nanoparticles using unbleached kraft fiber. Because gold atoms are more electronegative and noble than silver atoms, they are expected to have the lowest oxidation state. As a result, previous research used lignin composted in unbleached cellulose as a more potent reducing agent. To broaden the scope of the study to lower electronegative noble metal, the possibility of synthesizing AgNPs from cellulose fiber was investigated using chemical composition from bleached kraft fiber. We proposed a simple *in situ* process for reducing and immobilizing AgNPs on the surface of cellulose fiber using a one-pot hydrothermal synthesis. UV–Vis diffuse reflectance spectroscopy (DRS), field-emission scanning electron microscopy (FE-SEM), and high-resolution X-ray photoelectron spectroscopy (XPS) were used to characterize the structure and properties of cellulose-AgNPs composites. The potential application of as-prepared cellulose-silver nanoparticle composite sheets for antibacterial packaging was investigated. Quantitative methods were used to evaluate the antibacterial activity of cellulose-AgNPs nanocomposite sheets against Gram-negative *Escherichia coli (E.coli)*.

## Experimental

### Materials

American Chemical Society (ACS) reagent-grade silver nitrate (AgNO_3_) was used as received from Sigma Aldrich (St. Louis, USA) without any further purification. Hard wood bleached kraft (HWBK) pulp was obtained in dry form from Dongil Paper (Ansan, Korea). Ultra-pure water with a specific resistivity of 18 MΩ·cm was used in this study.

### Disintegration and Refining of Pulp

Dry HWBK pulp (350 g) was thoroughly soaked overnight in 5 L of water at room temperature. The fiber was disintegrated with a valley beater before being beaten to ~450 mL Canadian standard freeness (CSF) according to TAPPI test methods, TAPPI T200 sp-01 (58) and TAPPI T227 om-04 (59).

### *In situ* Synthesis of Cellulose-AgNP Composite Fiber

The synthesis concept was adapted from previous research (4, 26). Various concentrations of AgNO_3_ solution (50, 100, 250, and 500 mM) were prepared as precursors for AgNPs at room temperature. The cellulose slurry was prepared by dissolving the HWBK fiber in deionized (DI) water (5% consistency) and stirring for 10 min. Next, 45 mL of well-dispersed cellulose slurry (represented to 2.25 g dried pulp) was poured into a conical tube and centrifuged at 3,000 rpm for 5 min. This cellulose fiber was ready for synthesis after the water was drained. Further, 1 and 3 mL of each AgNO_3_ solution were mixed with 29 and 27 mL of DI water, respectively, and added to the as-prepared cellulose fiber. The effects of the amount and concentration of the silver precursor on the obtained AgNP-impregnated cellulose fibers were investigated by running the reaction for 30 min in an autoclave at 101.35 kPa and 132°C.

### Preparation of Cellulose-AgNP Composite Sheets

To prepare cellulose-AgNP composite sheets, 0.15 g moist composite fibers were mixed with 300 mL of DI water (0.05% consistency). Subsequently, the mixture was vacuum-suctioned onto a 200-mesh wire to form a wet sheet, which was then vacuum-dried for 8 h at 60°C. A paper made entirely of HWBK pulp (denoted as blank) was also prepared and used as a reference.

### Characterization of Cellulose-AgNP Composite

UV-Vis DRS spectra of as-prepared cellulose-AgNP composite sheets were obtained at 300–800 nm using a double-beam V-600 spectrophotometer (Jasco, Japan) with an attached integrating sphere.

Surface sputtering with platinum/palladium was performed on the specimen for 10 s using a Cressington Sputter Coater 108 auto (Cressington Scientific Instruments, UK). Field emission scanning electron microscopy (FESEM-Quanta FEG 250, FEI, Oregon, USA) was used to examine the surface morphology of the composite sheet, as well as the particle size, shape, and dispersity of the as-obtained AgNPs at 6,000 and 24,000 magnifications.

X-ray photoelectron spectroscopy (XPS, AXIS-NOVA, Kratos Analytical Ltd., UK) was used to investigate the chemical composition and bonding state of the silver created in/on the cellulose-AgNP composite. XPS spectra were collected using a hemispherical analyzer equipped with an Al K X-ray source (hv = 160 eV) and operating in an ultra-high vacuum chamber, chamber, 2.0 × 10^−9^ Torr.

### Antimicrobial Activity of Cellulose-AgNP Composite Sheet

The antimicrobial activity of the as-prepared cellulose-AgNP composite sheet was quantitatively tested according to the JIS Z 2801: 2000 standard. A single colony of *Escherichia coli (E. coli)* ATCC 8739 was transferred into a sterilized liquid nutrient broth medium and incubated for 24 h in an incubator at 37°C and 90% relative humidity. The initial population of inoculated E.coli was ~6 × 107 colony-forming units (CFU) per ml. Following this, the specimen (1 ×1 cm), which had been sterilized under ultraviolet light for ~1 h, was incubated in a suspension of inoculated *E. coli*. After 24 h, serial dilutions of the inoculum were cultured on MacConkey agar plate and incubated at 37°C for 24 h.

The number of bacteria colonies recovered on the agar plate was counted, and the percent reduction of bacteria (R) was calculated using Equation 1.


(1) $$\begin{array}{r} R\;\left(\%\right)=\frac{\left(B-C\right)}{B}\;\times 100 \end{array}$$

Where *B* represents the *E. coli* CFU from the control after 24 h and *C* represents the *E. coli* CFU from the cellulose-AgNP composite sheet after 24 h incubation.

## Results and Discussions

**Figure 1:**
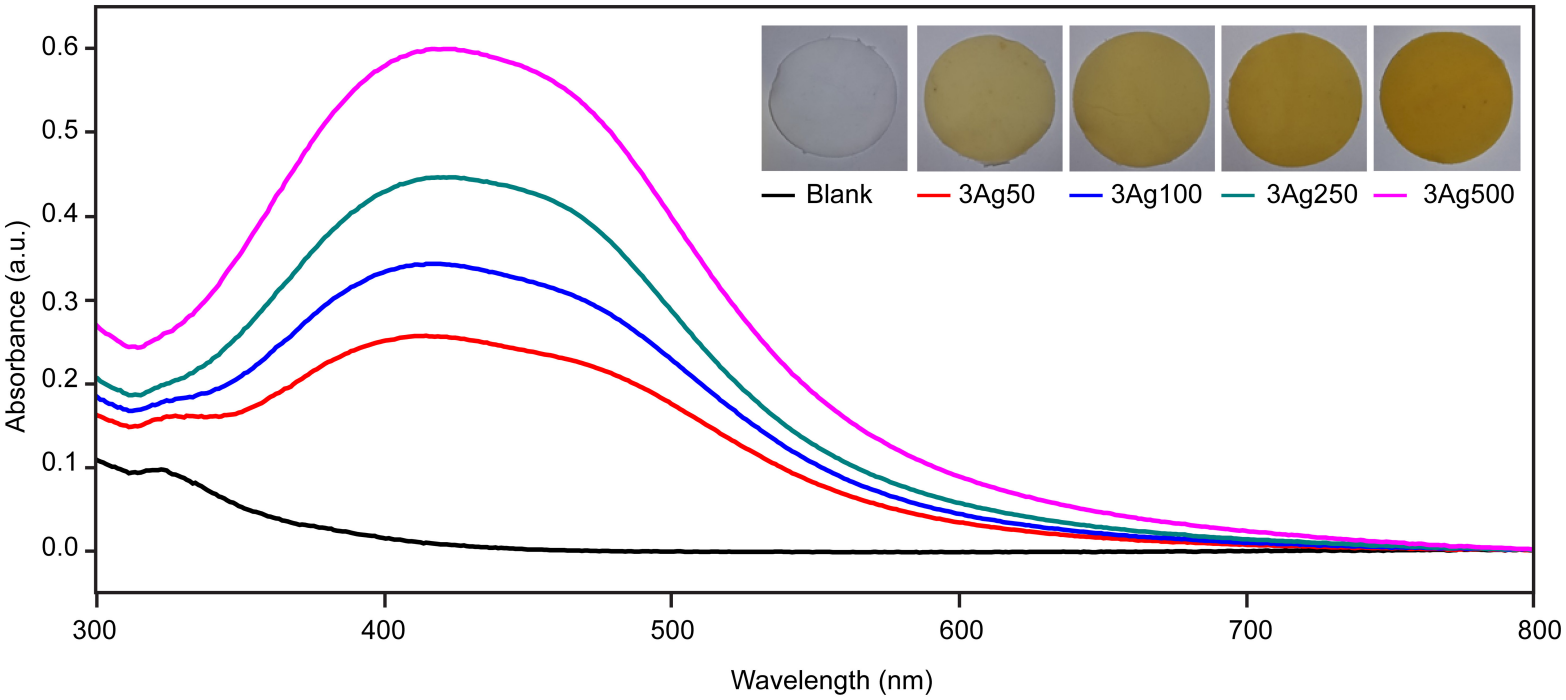
Spectra of UV–Vis diffuse reflectance of cellulose fiber-AgNP composite sheets. Digital photos of as-prepared cellulose fiber-AgNP composite sheets are shown in the inset figures.

This study developed a simple green reduction method for preparing cellulose-AgNP composite by using HWBK pulp. According to theory, AgNPs have a pale yellow to dark brown color due to surface plasmon resonance (SPR) excitation (60); AgNP formation was confirmed by UV-vis spectroscopy, as shown in Figure 1. UV-Vis spectra of blank and cellulose-AgNP composite sheets from various AgNO_3_ concentrations were obtained. The maximum absorbance occurs in the range of 410–415 nm regardless of molar concentration, but the intensity steadily increases as a function of molar concentration. This absorbance range is a characteristic of AgNPs caused by the excitation of longitudinal plasmon vibrations (61). Furthermore, the corresponding color of the cellulose-AgNP composite sheets, which transformed from opaque white to yellow, is shown in the inset of Figure 1. The 3Ag500 composite sheet with the highest absorbance intensity produced the darkest yellow. The results indicate that the concentration of silver precursor influenced the formation of AgNPs by enhancing the oxidation of cellulose hydroxyl groups. However, because the resonant frequency represented by absorbance did not shift, it was expected that these factors would have no effect on the shape and size of the as-obtained AgNPs (60).

**Figure 2:**
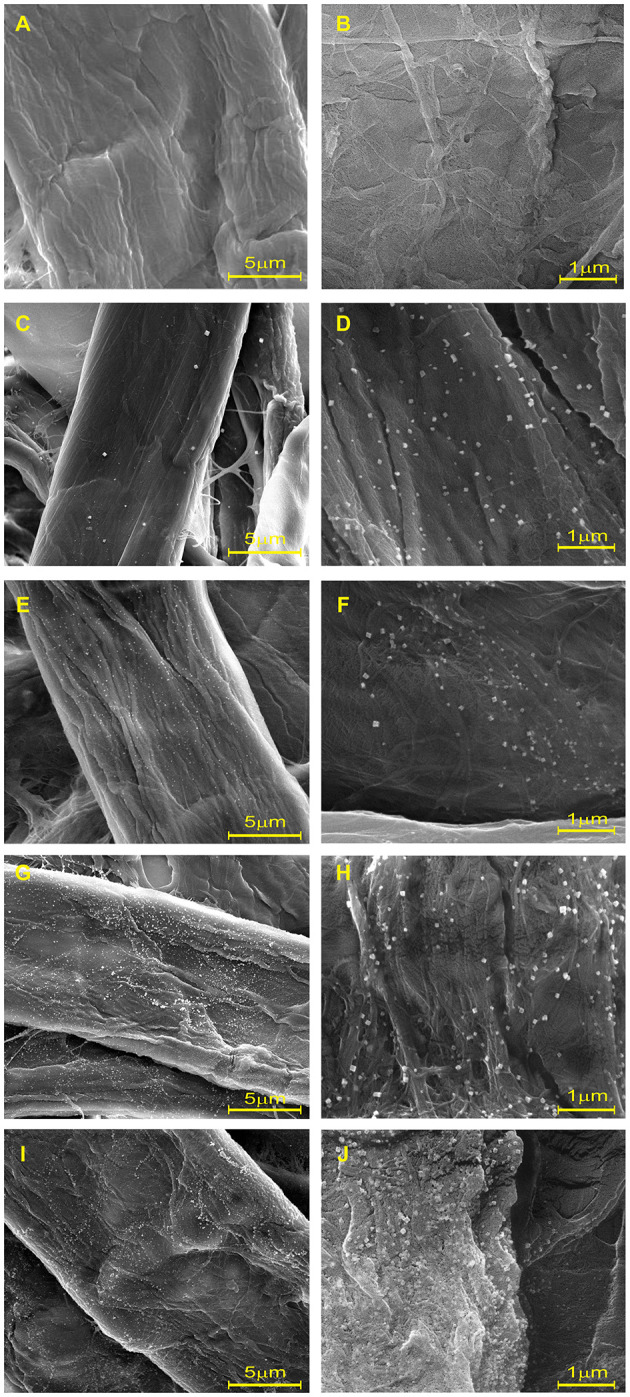
Scanning electron microscopy at 6,000× and 2,4000× of **(A,B)** blank and cellulose-AgNP composite sheets: **(C,D)** 3Ag50, **(E, F)** 3Ag100, **(G,H)** 3Ag250, and **(I,J)** 3Ag500.

To investigate the formation and distribution of AgNPs on cellulose fiber, the nanocomposite sheets were examined and compared with blank sheets *via* FE-SEM, as shown in Figure 2. The smoothness and cleanliness of the cellulose surface, as seen from the blank sheet, are depicted in Figures 2A,B. In contrast, Figures 2C–J show that the *in-situ* reduction and immobilization method successfully produced AgNPs as white dots dispersed across the fiber surface. The AgNPs uniformly and evenly adhered to the surface of the fibers. The number and coverage of nanoparticles on the cellulose fibers increased as the precursor concentration increased. Regardless of the molar concentration of AgNO_3_, the morphology of the synthesized AgNPs was clearly observed in a cubic shape at 2,4000X magnification. This finding is consistent with the higher intensity of SPR spectra in UV-Vis absorbance without peak shift, as shown in Figure 1.

**Figure 3:**
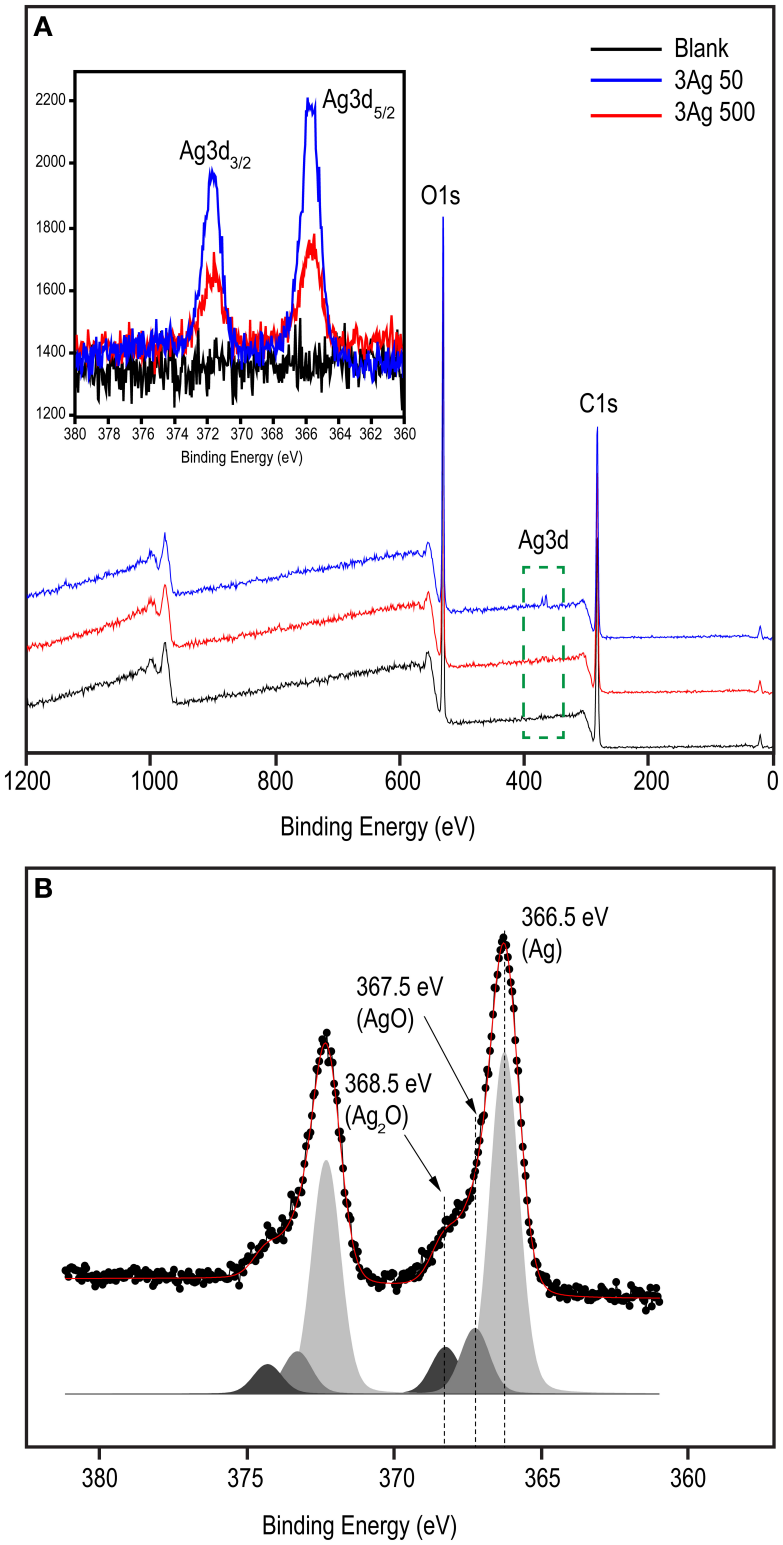
XPS spectral analysis of blank and cellulose-AgNP composite sheets showing **(A)** survey scan and **(B)** corresponding deconvoluted peaks in the high resolution (HR) spectra of Ag3d. Inset shows the Ag3d binding energy spectra.

XPS analysis was performed to investigate the chemical state of the cellulose-AgNP composite. The survey spectra in Figure 3A clearly show the presence of carbon (C1s) and oxygen (O1s) from cellulose in both the blank and nanocomposite sheets. As shown in the inset of Figure 3A, the XPS spectra clearly reveal the elemental status of Ag3d, which are doublet peaks formed by spin–orbital coupling; Ag3d5/2 (365.70 eV), and Ag3d3/2 (371.50 eV) (62). A high-resolution analysis of Ag3d was performed for further investigation, and the core-level spectrum is shown in Figure 3B. The spectrum deconvolutes into three components with binding energies of 366.5 eV (Ag^0^), 367.5 eV (AgO), and 368.5 eV (AgOH) (Ag_2_O). This result clearly demonstrates that silver predominately exists in the metallic form (Ag0), indicating the formation of AgNPs on the surface of the cellulose fiber.

**Figure 4:**
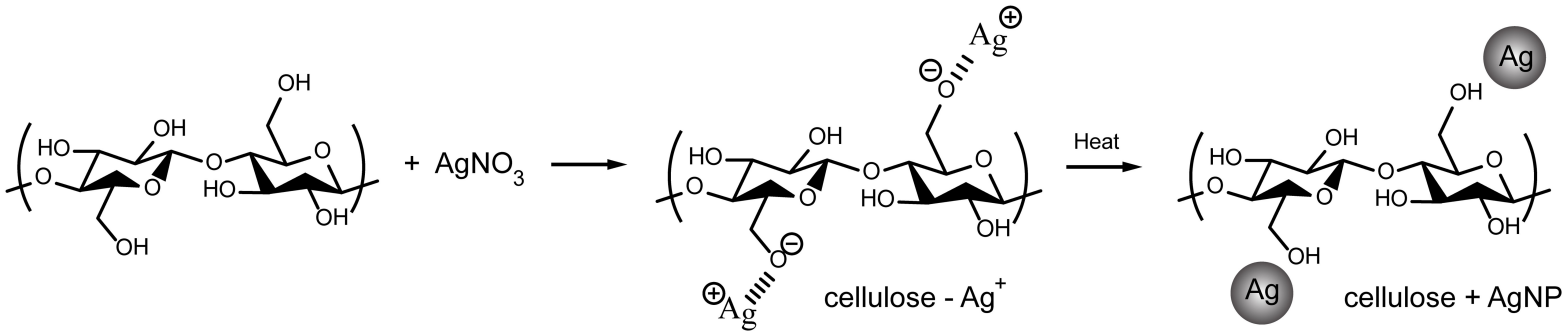
Schematic diagram depicting possible synthetic mechanisms for cellulose-AgNPs composites.

A large number of polar hydroxyl and ether groups on the cellulose polymer chain plays an important role in the synthesis of AgNPs, as illustrated in Figure 4. They are expected to interact with silver cations (Ag^+^) *via* electrostatic (i.e., ion-dipole) interactions. Subsequently, the silver cations are reduced and transformed into silver atoms (Ag0), which are agglomerated into oligomeric clusters (63). These clusters eventually result in the formation of nanoparticles and their simultaneous immobilization on the cellulose surface, causing the cellulose color to change from white to yellow. Mild reducing agents, such as hydroxyl groups, generally require a catalyst or additional energy to be activated (64); therefore, heat from the hydrothermal process is used to accelerate this reaction. The simultaneous immobilization of nanoparticles provides an important advantage to nanocomposites because it can suppress undesirable phenomena, such as nanoparticle aggregation and modification, after synthesis. Furthermore, the active hydroxyl groups may be capable of driving silver oxide precipitation from soluble silver salt solutions. However, the obtained silver oxide can be thermally decomposed to yield AgNPs (65). As a result, it is possible that silver oxide (AgO and Ag2O) was formed by a side reaction, as observed in the XPS spectra (Figure 3), but it was reduced by decomposition to AgNPs.

**Figure 5:**
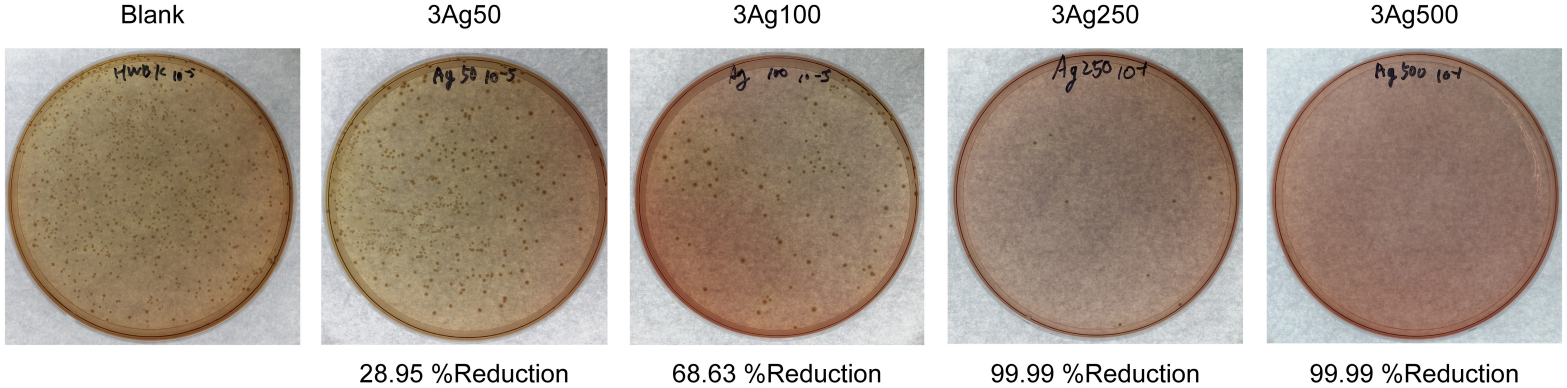
Bactericidal effect of cellulose-AgNPs composite sheets on colony-forming unit against gram-negative bacteria *E. coli* at various levels of precursor concentration.

The antibacterial activity of the cellulose-AgNPs composite sheets against *E. coli* bacteria was investigated. Figure 5 depicts the effect of composite sheets made from various molar concentrations of silver precursor on bacterial growth reduction. The cellulose-AgNPs composite sheets derived from a lower molar concentration of silver precursor were able to inhibit bacterial growth to some extent, with fewer bacterial colonies formed on the agar plates when compared to the blank. However, the bacteria were completely killed in cellulose-AgNPs composite sheets containing higher molar concentrations of silver precursor, such as 3Ag250 and 3Ag500. This is clearly demonstrated by the reduction rate of ~99.99%. Gram-negative bacteria have an outer membrane outside the peptidoglycan layer that acts as a selective permeability barrier, protecting bacteria from harmful agents such as detergents, drugs, and degradative enzymes while also allowing nutrients to penetrate and sustain bacterial growth. Previous research has suggested that the mechanism underlying the bactericidal properties of AgNPs is electrostatic repulsion or attraction. The bacterial surface is negatively charged due to carboxylic and phosphonate groups in the outer membrane. As a result, the cell wall quickly binds to Ag+ ions discharged from AgNPs followed by interacting with the ribosome and inhibit the expression of enzymes and proteins required for ATP production, thereby, eventually killing *E. coli* bacteria (66, 67). This finding was consistent with the findings of Amini et al. (40), who investigated the effect of paper packaging coated with AgNPs-impregnated cellulose nanofiber on *E. coli*. The higher the concentration of AgNPs on the surface of cellulose fiber, the better the antimicrobial activity.

## Conclusion

Cellulose-AgNP composites were successfully prepared using different molar concentrations of AgNO_3_ in a one-step direct synthesis with HWBK fiber and tested for antimicrobial activity against gram-negative *E. coli*. The advantages of this synthetic method are its simplicity and that it uses no external chemicals for reduction, immobilization, and stabilization of AgNPs. UV-Vis DRS and XPS analyses revealed that AgNPs were successfully bioreduced to metallic Ag from Ag^+^. The FE-SEM micrograph clearly demonstrated that the AgNPs were well-conjugated directly onto the cellulose fiber surface. The AgNP imparts antimicrobial properties to the cellulose fiber, which has been successfully demonstrated against the food-borne pathogenic bacterium gram-negative *E. coli*. The cellulose-AgNP composite sheets with low molar concentrations of silver can achieve a certain antimicrobial activity, whereas *E. coli* growth was completely inhibited with a 99.99% reduction at the 3Ag250 and 3Ag500 conditions. These findings suggest that the cellulose fiber based AgNP nanocomposite presented in this study may be an alternative antibacterial paper for active food packaging.

## Data Availability Statement

The original contributions presented in the study are included in the article/supplementary material, further inquiries can be directed to the corresponding author/s.

## Author Contributions

SKw: methodology, investigation, and manuscript preparation. WL: methodology, experiment, and investigation. JC: conceptualization, methodology, and investigation. NB: research and manuscript preparation. SKo: conceptualization, funding acquisition, methodology, supervision, validation, and manuscript–reviewing and editing. All of the authors listed have made a significant, direct, and intellectual contribution to the work and have given their permission for it to be published.

## Conflict of Interest

The authors declare that the research was conducted in the absence of any commercial or financial relationships that could be construed as a potential conflict of interest.

## Publisher's Note

All claims expressed in this article are solely those of the authors and do not necessarily represent those of their affiliated organizations, or those of the publisher, the editors and the reviewers. Any product that may be evaluated in this article, or claim that may be made by its manufacturer, is not guaranteed or endorsed by the publisher.
